# Economic Impact of Lyme Disease

**DOI:** 10.3201/eid1204.050602

**Published:** 2006-04

**Authors:** Xinzhi Zhang, Martin I. Meltzer, César A. Peña, Annette B. Hopkins, Lane Wroth, Alan D. Fix

**Affiliations:** * Centers for Disease Control and Prevention, Atlanta, Georgia, USA;; † University of Maryland, Baltimore, Maryland, USA;; ‡ Care First-Easton Branch (previously Delmarva Health Plan), Easton, Maryland, USA

**Keywords:** Vector-borne disease, Lyme disease, cost of illness, hospital charge, research

## Abstract

To assess the economic impact of Lyme disease (LD), the most common vectorborne inflammatory disease in the United States, cost data were collected in 5 counties of the Maryland Eastern Shore from 1997 to 2000. Patients were divided into 5 diagnosis groups, clinically defined early-stage LD, clinically defined late-stage LD, suspected LD, tick bite, and other related complaints. From 1997 to 2000, the mean per patient direct medical cost of early-stage LD decreased from $1,609 to $464 (p<0.05), and the mean per patient direct medical cost of late-stage LD decreased from $4,240 to $1,380 (p<0.05). The expected median of all costs (direct medical cost, indirect medical cost, nonmedical cost, and productivity loss), aggregated across all diagnosis groups of patients, was ≈$281 per patient. These findings will help assess the economics of current and future prevention and control efforts.

## Abstract 2

Since 1975, Lyme disease has become the most common vectorborne inflammatory disease in the United States.

Lyme disease (LD) is a multisystem, multistage, inflammatory tickborne disorder caused by the spirochete *Borrelia burgdorferi*. LD usually begins with an initial expanding skin lesion, erythema migrans (EM), which may be followed by musculoskeletal, neurologic, and cardiac manifestations in later stages of the disease (*1*–*3*). Enzyme-linked immunosorbent assay and Western blotting test are widely used to diagnose LD (*4*–*6*). LD is most responsive to antimicrobial drugs in the early stage, while further intensive therapy may be necessary in the late stage (*7*,*8*). A variety of prevention and control procedures can be implemented to prevent and reduce LD incidence, including, but not limited to, public education; personal protection measures such as wearing protective clothing (gloves, long clothes), checking one's body daily for ticks, avoiding tick-infested areas, and applying tick repellent (DEET, permethrin); host management; habitat modification; and chemical control (*9*,*10*). In 1998, the Food and Drug Administration approved a recombinant outer-surface protein A (rOspA) LD vaccine (LYMErix, SmithKline Beecham Biologicals, Rixensart, Belgium) for persons 15–70 years of age (*11*). However, in 2002, SmithKline withdrew the vaccine, citing low demand. Therefore, personal protection measures, early diagnosis, and early treatment are extremely important in preventing and controlling LD.

Since the first case reported in 1975 (*12*), LD has become the most common vectorborne inflammatory disease in the United States. Foci of LD are widely spread in the northeastern, mid-Atlantic, and north-central regions of the United States (*13*). Despite federal, state, and local efforts to prevent and control LD, total reported cases of LD increased almost 3-fold from 1991 to 2002 (Figure 1). In 2002, the Centers for Disease Control and Prevention (CDC) received reports of 23,763 LD cases, 95% of which were from Connecticut, Delaware, Maine, Maryland, Massachusetts, Minnesota, New Hampshire, New Jersey, New York, Pennsylvania, Rhode Island, and Wisconsin (*14*). In Maryland, the overall incidence of LD was more than twice as high as the overall incidence of LD in the United States (13.0 vs. 6.3 cases per 100,000 population) (*13*).

**Figure 1:**
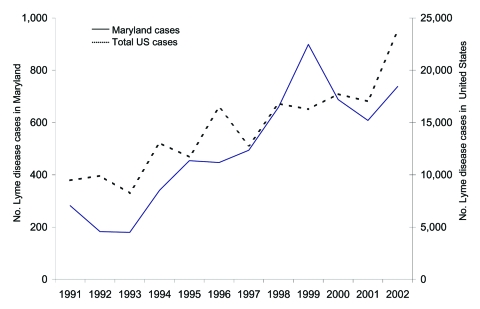
Lyme disease (LD) cases reported to the Centers for Disease Control and Prevention by state health departments in the United States (1991–2002). Reported cases were defined according to the national surveillance definition. For the purpose of surveillance, a case of LD is defined as physician-diagnosed erythema migrans >5 cm or >1 late rheumatologic, neurologic, or cardiac manifestation with laboratory evidence of *Borrelia burgdorferi* infection. Available from http://www.cdc.gov/ncidod/dvbid/lyme/epi.htm (*14*).

Assessing the economic impact of LD will help assess the economics of current and future prevention and control efforts. Although several studies of cost estimates of LD have been published (e.g., *15*), information on the economic impact of LD is limited. Therefore, we conducted a 4-year study to estimate the economic impact of LD on the Maryland Eastern Shore.

## Methods

### Study Population and Data

This study was conducted in 5 counties (Caroline, Dorchester, Kent, Queen Anne, and Talbot) on the Maryland Eastern Shore, an area where LD is endemic (Table 1). The study population includes patients living in the 4 counties enrolled in Delmarva Health Plan (DHP, a managed healthcare organization) and non-DHP patients receiving health care from office-based physicians in Kent County from 1997 to 2000. Eligible patients were identified through records of encounters for LD, tick bites, insect bites, and serologic testing for LD antibodies. During 1997 and 1998, identified patients were contacted for informed consent. Patients who indicated that they did not wish to participate were excluded from our database. A cost and risk questionnaire (Appendix 1) was sent to patients who gave informed consent. The response rate of the survey was ≈22%. Interviewers then reviewed patients' charts and consulted relevant sources (e.g., hospital, physician office, laboratory) to obtain the following information: patient demographics; insurance coverage; diagnosis; symptoms; dates of onset and diagnosis; dates of tick bite exposure; dates and costs of primary provider and consultant visits; dates and costs of hospitalizations and emergency department visits; dates, results, and costs of laboratory tests; and dates and costs of antimicrobial drug treatment. All abstracted information was kept confidential. After 1999, an anonymous abstraction of medical records was approved by the institutional review board (IRB) and implemented, allowing inclusion of more patients for all 4 study years, with the exclusion of the records of those who had previously declined participation. All protocols of this study were approved by IRBs from CDC, the state of Maryland, and the University of Maryland. Those patients identified as having received an LD vaccination were not included in this study.

**Table 1:** Reported cases* of Lyme disease (LD) in Maryland Eastern Shore, 1997–2000†

County	1997	1998	1999	2000	Total
Caroline	18	17	26	21	82
Dorchester	3	4	3	4	14
Kent	24	47	20	34	125
Queen Anne	32	31	40	35	138
Talbot	13	22	33	37	105
Total	90	121	122	131	464

*Reported cases defined according to the national surveillance definition. For the purpose of surveillance, a case of LD is defined as physician-diagnosed erythema migrans >5 cm or >1 late rheumatologic, neurologic, or cardiac manifestation with laboratory evidence of *Borrelia burgdorferi* infection. †Source: Maryland Department of Health and Mental Hygiene. Available from http://www.edcp.org/vet_med/lyme_disease.html

### Case Definition

For the purpose of surveillance, a case of LD is defined as physician-diagnosed EM >5 cm or at least 1 late rheumatologic, neurologic, or cardiac manifestation with laboratory evidence of *B. burgdorferi* infection (*16*). These criteria were developed as an epidemiologic case definition intended for surveillance purposes only. Although such a standard may aid comparison across clinical studies and facilitate development of research, exposure history and clinical features are critical. For example, treating patients with seasonal (summer) musculoskeletal flulike symptoms in areas where LD is endemic may be clinically appropriate (*12*). Because the data for this study were collected directly from healthcare organizations and physicians, we used a clinical definition of LD. This definition was based on physicians' determination in the medical record, according to patients' clinical findings, tick exposure, and other relevant details (e.g., laboratory results).

In our study, LD patients were identified by using a final diagnosis code in their medical records. LD patients were then divided into 5 diagnosis groups: clinically defined early-stage LD, clinically defined late-stage LD, suspected LD, tick bite, and other related complaints. Most clinically defined early-stage LD patients had EM; some also had musculoskeletal flulike symptoms such as malaise, fatigue, headache, fever, and chills (*12*). In this study, clinically defined late-stage LD patients included those with later manifestations (neurologic involvement, cardiac involvement, and arthritis) and patients with chronic LD. The diagnosis groups of suspected LD, tick bite, and other related complaints involved all patients without a clear final diagnosis of LD. Suspected LD referred to patients who had some symptoms that could be indicative of LD without further evidence and thus no definitive diagnosis of LD. Patients with tick bites without symptoms were placed in the tick bite group. The diagnosis group of other related complaints included all other diagnoses that were different from the above 4 diagnosis groups, such as unknown insect bites and screening among asymptomatic persons.

### Study Design

We calculated the following total costs of LD: 1) direct medical costs of LD diagnosis and treatment, 2) indirect medical costs, 3) nonmedical costs, and 4) productivity losses. Intangible costs (e.g., costs incurred because of pain and suffering) were not incorporated. Consumer price index (CPI) for medical care was used to adjust all medical payments into year 2000 dollars (*17*). For nonmedical costs and productivity losses, we adjusted costs by using the general CPI. We took a societal perspective, which incorporates all costs and all benefits no matter who pays costs or who receives benefits.

Charges were used to estimate the direct medical cost. To determine the direct medical costs associated with LD, we used charge data from both DHP and office-based healthcare providers in Kent County. Direct medical costs of LD included costs (charges) of physician visits, consultation, serology, procedure, therapy, hospitalization/emergency room (ER), and other related costs (Appendix 2).

Indirect medical costs, nonmedical costs, and productivity losses were all acquired from a patient questionnaire used in 1997 and 1998. The questionnaire was sent to LD patients with informed consent forms. Collection of these data was restricted to those 2 years. In this study, indirect medical costs refer to extra prescription and nonprescription drug costs that patients paid out of pocket.

The patient's questionnaire also collected information on nonmedical payments made for home or health aides and miscellaneous services, such as travel (transportation) and babysitting. Each patient's transportation costs to a physician's office were estimated by using the US federal government reimbursement rate, multiplying the reported total travel miles per patient by $0.365/mile. Total travel mileage per patient was calculated by counting the number of physician visits and multiplying total visits by the distance of a round trip to the physician's office.

We used patient-reported time lost from work to estimate productivity losses due to LD on the basis of the human capital method and valued the time lost by using age- and sex-weighted productivity valuation tables (*18*). Because of the potential complexity of accurately answering the question, we did not ask patients to estimate the time they lost from household production. We did, however, ask patients if they paid anybody to do household tasks because their LD-related infirmities prevented them from doing those tasks. For patients <15 years of age, we assumed that their parents (usually the mother) had to take time off from their work to take care of them. Therefore, their mothers' values of lost days of work were included.

### Analysis

We used the following formula to estimate the average per capita cost of LD, i.e., the mean cost (direct medical costs, indirect medical costs, nonmedical costs, and productivity losses) aggregated across all diagnosis groups of patients:

Expected mean cost of a LD outcome = Σ direct medical costs, indirect medical costs, nonmedical costs, and productivity losses (Mean cost of outcome clinically defined early-stage LD, clinically defined late-stage LD, suspected LD, tick bite, and other related complaints × Probability of outcome clinically defined early-stage LD, clinically defined late-stage LD, suspected LD, tick bite, and other related complaints).

Because the distribution of cost data is often not normal, we also calculated the medians of these costs and used both mean and median to estimate the most likely per capita cost of LD on the Maryland Eastern Shore. The median cost of an LD outcome was calculated by using the following formula:

Expected median cost of a LD outcome = Σ direct medical costs, indirect medical costs, nonmedical costs, and productivity losses (Median cost of outcome clinically defined early-stage LD, clinically defined late-stage LD, suspected LD, tick bite, and other related complaints × Probability of outcome clinically defined early-stage LD, clinically defined late-stage LD, suspected LD, tick bite, and other related complaints)

Differences between annual mean direct medical costs were analyzed by using 1-way analysis of variance followed by a Bonferroni test. Differences were considered significant for p values <0.05. Additionally, we used a multivariate linear regression model to estimate the relative impact of a number of factors on the direct medical costs of LD. The ordinary linear regression (OLS) method was applied by using SAS 8.2 (SAS Institute, Cary, NC, USA) and Stata SE (StataCorp LP, College Station, TX, USA). The dependent variable was total direct medical cost per LD patient. We transformed total direct medical costs by using natural logarithms because the data were highly skewed. Independent variables of the equation included cohort year, LD diagnosis groups, diagnostic and treatment procedures, and patient characteristics (e.g., sex, age). All independent variables, except age, were binomial (yes = 1, no = 0). Baseline costs (i.e., the intercept term in the regression equation) referred to those costs accrued by a woman who had tick bite only (without EM symptoms) diagnosed in 1997 during an office visit. Such a patient had no hospital or ER stay, no serologic tests, no consultation from other physicians, no antimicrobial drug therapy, and no other procedures outside a physician office and hospital/ER. Additional direct medical costs were added or subtracted to the baseline costs for each independent variable of interest if significant (Appendix 3). We tested heteroscedasticity in Stata and corrected mild heteroscedasticity by using "robust" and "hc3" procedures. We also tested both linearity and multicollinearity in SAS and Stata.

## Results

From 1997 to 2000, we identified 3,415 LD-relevant patients in the 5 counties studied on Maryland Eastern Shore (Table 2). Among them, 10% had clinically defined early-stage LD while almost 5% of all patients had clinically defined late-stage LD. Of 284 patients who returned a completed patient questionnaire, 59 patients had clinically defined early-stage LD; 25 patients had clinically defined late-stage LD.

**Table 2:** Distribution of Lyme disease (LD) cases* in Maryland Eastern Shore, 1997–2000

Diagnosis group†	No. LD cases (%) from medical record abstraction‡	No. LD cases (%) from follow-up patient survey§
Early stage	334 (10)	59 (21)
Late stage	156 (5)	25 (9)
Suspected LD	718 (21)	54 (19)
Tick bite	539 (16)	62 (22)
Other	1,668 (49)	84 (30)
Total	3,415 (100)	284 (100)

*LD cases in the study are clinically defined LD cases, which may not fit surveillance definition because the data were collected directly from healthcare organizations and physicians. †Patients were divided into 5 diagnosis groups: clinically defined early-stage LD, clinically defined late-stage LD, suspected LD, tick bite, and other related complaints. ‡Number of patients (1997–2000) who were identified through records of encounters for LD, tick bites, insect bites, and serologic testing. §Number of patients (1997–1998) who answered a questionnaire recording indirect medical costs, nonmedical costs, and productivity losses.

Table 3 provides cohort years, medians, means, and standard deviations of direct medical costs comparing the different diagnosis groups. During the study's time frame, the mean (range) direct medical cost of clinically defined early-stage LD decreased from $1,609 ($95–$11,286) in 1997 to $464 ($5–$5,338) in 2000 (p<0.05). The mean direct medical cost of clinically defined late-stage LD decreased from $4,240 ($275–$24,985) in 1997 to $1,380 ($45–$6,918) in 2000 (p<0.05).

**Table 3:** Summary of direct medical cost*† per Lyme disease (LD) patient in Maryland Eastern Shore, 1997–2000

Diagnosis group‡	Cohort	No. cases	Cost per case (US$)					Significance§			
			Median	Mean	Minimum	Maximum	SD	1997	1998	1999	2000
Early-stage LD	1997	77	565	1,609	95	11,286	2,010	NA			
	1998	63	337	869	78	9,720	1,542	S	NA		
	1999	122	282	455	42	3,574	630	S	NS	NA	
	2000	72	288	464	5	5,338	738	S	NS	NS	NA
Late-stage LD	1997	28	3,673	4,240	275	24,985	5,132	NA			
	1998	24	654	1,472	125	6,417	1,839	S	NA		
	1999	59	588	1,286	74	5,402	1,334	S	NS	NA	
	2000	45	589	1,380	45	6,918	1,652	S	NS	NS	NA
Suspected LD	1997	153	169	326	45	9,564	948	NA			
	1998	79	174	255	48	2,285	281	NS	NA		
	1999	242	198	321	51	3,869	445	NS	NS	NA	
	2000	244	238	361	42	7,816	601	NS	NS	NS	NA
Tick bite	1997	143	92	140	33	836	129	NA			
	1998	55	93	227	34	3,432	502	S	NA		
	1999	202	87	120	17	527	98	NS	S	NA	
	2000	139	70	121	16	1,181	141	NS	S	NS	NA
Other	1997	490	196	319	8	6,236	495	NA			
	1998	154	273	479	34	3,721	561	S	NA		
	1999	573	215	321	36	5,091	435	NS	S	NA	
	2000	451	256	381	17	4,157	452	NS	NS	NS	NA

*Direct medical costs were collected from medical record abstraction (1997–2000). Direct medical costs of LD included costs of physician visits, consultation, serologic testing, procedure, therapy, hospitalization/ER, and other relevant costs. †All costs were converted to 2000 equivalent. ‡Patients were divided into 5 diagnosis groups: clinically defined early-stage LD, clinically defined late-stage LD, suspected LD, tick bite, and other related complaints. §Differences between annual mean direct medical costs were analyzed by using 1-way analysis of variance followed by Bonferroni test; p<0.05; SD, standard deviation; NA, not available; S, significant; NS, not significant.

**Figure 2:**
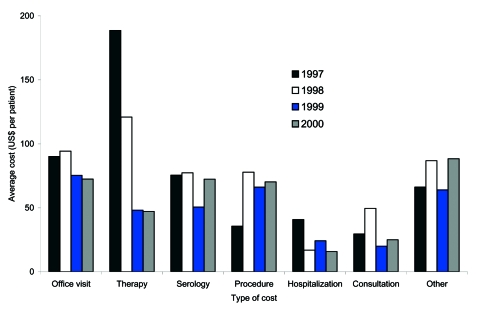
Distribution of elements of direct medical cost (US$) per Lyme disease (LD) patient in Maryland Eastern Shore (1997–2000). Mean is based on direct medical costs of LD patients. Direct medical costs were collected from medical record abstraction (1997–2000). Direct medical costs of LD included costs of physician visits, consultation, serologic tests, procedure, therapy, hospitalization/emergency room, and other relevant costs. All costs were converted to 2000 equivalent.

From 1997 to 2000, the mean cost of therapy of all diagnosis groups decreased 75%, from $189 to $47, and the mean cost of hospitalization/ER decreased 61%, from $41 to $16 (Figure 2). During the same period, the mean cost of an office visit, consultation, and serologic tests also decreased 20%, 15%, and 4%, respectively. Additionally, the proportion of patients within the highest percentile (95th percentile for all 4 years) of therapy cost gradually decreased from 8% in 1997 to 7% in 1998, to 4% in 1999, and 3% in 2000 (data available upon request).

A patient with clinically defined early-stage LD paid an average of $164 in 1997 and $307 in 1998 (in 2000 dollars) for extra prescription and nonprescription drugs (Table 4). Those with clinically defined late-stage LD paid, for similar items, an average of $579 in 1997 and $389 in 1998. The mean nonmedical cost for clinically defined early-stage LD was $109 in 1997 and $23 in 1998. For patients with clinically defined late-stage LD, mean nonmedical costs were $60 in 1997 and $6,703 in 1998. During the survey period, the mean productivity loss of clinically defined early-stage LD was $411 in 1997 and $88 in 1998, and the mean productivity loss of clinically defined late-stage LD was $7,762 in 1997 and $9,108 in 1998. For all 3 types of costs shown in Table 4, a large difference was seen between mean and median values, with the latter often less than half of the mean value, indicating that a small number of LD patients account for a large portion of total costs.

**Table 4:** Indirect medical cost, nonmedical cost, and productivity loss*† per Lyme disease (LD) patient in Maryland Eastern Shore, 1997–1998

Diagnosis group‡	Cohort	No.	Indirect medical cost (US$)§			Nonmedical cost (US$)¶			Productivity loss (US$)#		
			Median	Mean	SD**	Median	Mean	SD	Median	Mean	SD
Early-stage LD	1997	20	20	164	428	27	109	219	28	411	1,095
	1998	39	8	307	1,773	8	23	71	49	88	85
Late-stage LD	1997	6	35	579	1,295	22	60	85	273	7,762	17,458
	1998	19	11	389	1,448	37	6,703	22,405	46	9,108	28,284
Suspected LD	1997	22	5	25	49	8	24	37	26	83	164
	1998	32	0	12	22	4	12	17	44	109	197
Tick bite	1997	31	0	37	105	9	155	731	7	73	151
	1998	31	0	11	40	8	17	50	19	66	79
Other	1997	33	0	31	102	11	143	696	28	233	605
	1998	51	0	11	21	4	23	95	19	300	1,539

*Indirect medical costs, nonmedical costs, and productivity losses were acquired from patient questionnaire (1997–1998). †All costs were converted to 2000 equivalent. ‡Patients were divided into 5 diagnosis groups: clinically defined early-stage LD, clinically defined late-stage LD, suspected LD, tick bite, and other related complaints. §Indirect medical costs refer to prescription and nonprescription drug costs patients paid out of pocket. ¶Nonmedical costs are payments made for home/health aides and miscellaneous services, such as transportation and babysitting. #Productivity losses refer to losses in earning due to illness. **SD, standard deviation.

Using multivariate linear regression analysis, we found that patients with clinically defined early- and late-stage LD had direct medical costs that were ≈50% and 100%, respectively, higher (p<0.001) relative to patients who only had tick bite, if the impact from other factors was not considered (Table 5). Moreover, patients who were hospitalized or made ER visits, who underwent serologic testing, who needed therapy, who were referred for consultation, and who had other procedures had substantially (p<0.001) higher direct medical cost than those who did not (Table 5). No cost difference was seen between men and women. After controlling for other factors, direct medical costs per LD patient in 2000 were lower than those in 1997 (Table 5).

**Table 5:** Impact on direct medical cost* due to cohort year, Lyme disease (LD) diagnosis groups, diagnostic and treatment procedures, and patient characteristics in Maryland Eastern Shore (regression results, n = 3,415)

	Direct medical cost (US$)	5th CI† (US$)	95th CI (US$)	p
Baseline cost‡	60.88	55.94	66.26	<0.0001
Additional direct medical cost§				
Clinically early stage	34.93	22.59	50.65	<0.0001
Clinically late stage	67.05	45.57	94.97	<0.0001
Suspected LD	3.16	-0.68	7.96	0.171
Other LD-relevant complaint	8.33	4.28	13.29	<0.0001
Serologic test¶	38.27	28.20	50.59	<0.0001
Procedure#	26.13	17.68	36.58	<0.0001
Hospitalization/emergency room (ER)**	114.96	89.85	145.83	<0.0001
Consultation††	84.68	68.09	104.56	<0.0001
Therapy‡‡	36.66	29.15	45.56	<0.0001
Miscellaneous§§	46.96	38.21	57.27	<0.0001
Erythema migrans¶¶	-9.56	-13.02	-4.90	<0.0001
Male	-0.68	-2.72	1.84	0.571
Each year of age##	0.11	0.05	0.19	<0.0001
Year 1998	-5.05	-9.28	0.54	0.0003
Year 1999	-12.74	-15.11	-9.50	0.0371
Year 2000	-9.09	-12.09	-5.08	<0.0001

*Direct medical costs of LD included costs of physician visits, consultation, serologic testing, procedure, therapy, hospitalization/ER, and other relevant costs. Patients were divided into 5 diagnosis groups: clinically defined early-stage LD, clinically defined late-stage LD, suspected LD, tick bite, and other related complaints. All costs were converted to 2000 equivalent. †CI, confidence interval. ‡Baseline costs refer to those costs accrued by a female patient who had tick bite only (with no erythema migrans symptoms), diagnosed in 1997 during an office visit. She had no hospital or ER stay, no serologic tests, no consultation, no therapy, and no other procedures (R^2^ = 0.67). §Additional direct medical costs are added or subtracted to the baseline costs for each variable of interest if significant (see Appendix 3 for details). ¶Serologic test (yes = 1, no = 0) refers to patients who had serologic test (e.g., enzyme-linked immunosorbent assay or Western blotting test). #Procedure (yes = 1, no = 0) refers to patients who had other procedures that were not performed in hospital/ER, consultation, or physician office. **Hospitalization/ER (yes = 1, no = 0) refers to patients who had hospital or ER stay. ††Consultation (yes = 1, no = 0) refers to patients who received consultation from other physicians. ‡‡Therapy (yes = 1, no = 0) refers to patients who had therapy charges including antimicrobial agents and additional costs associated (e.g., registered nurse home visits). §§Miscellaneous (yes = 1, no = 0) refers to patients who had other appropriate charges such as charges for additional laboratory tests. ¶¶ Refers to patients with erythema migrans (yes = 1, no = 0). ##Age is a continuous variable and refers to each additional year of age of the patient.

**Figure 3:**
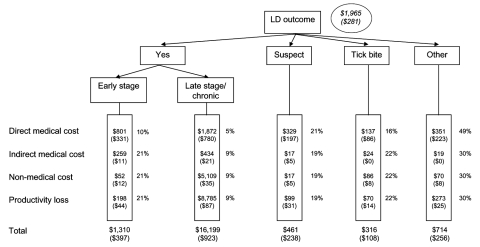
Expected mean (median) cost per Lyme disease (LD) patient in Maryland Eastern Shore by using LD outcome tree. Direct medical costs collected from medical record abstraction (1997–2000). Indirect medical costs, nonmedical costs, and productivity losses were acquired from patient questionnaire (1998–1999). The mean (median) of all costs was aggregated across all diagnostic groups of patients. Percentages refer to probabilities of outcome of a possible LD case (clinically defined early-stage LD, clinically defined late-stage LD, suspected LD, tick bite, and other related complaints). Total percentages do not add to 100% because of rounding. All costs were converted to 2000 equivalent.

In year 2000 dollars, the expected mean total cost attributable to LD was $1,965 per patient, and the expected median total cost attributable to LD was estimated at $281 per patient (Figure 3). For LD patients at the clinically defined early stage, the median total cost was ≈$397 (mean $1,310), whereas for patients at the clinically defined late stage, the median cost rose to $923 (mean $16,199). Suspected LD cases, tick bite cases, and other LD-related complaints had median costs of $238 (mean $461), $108 (mean $316), and $256 (mean $714), respectively.

## Discussion

Previous studies of the economic impact of LD were often based on numerous assumptions and experts' suggestions (e.g., Maes et al. [*15*]). Only a few studies provided cost estimates of LD based on data collected from the field (e.g., Fix et al. [*19*], Strickland et al. [*20*]). Even in those studies, however, cost estimates only related to direct medical charges or certain diagnosis or treatment procedures. By combining data from medical records with results from a patient survey, this study more comprehensively documents the economic impact of LD from a societal perspective.

To approximate the annual economic impact of LD nationwide, we extrapolated our results to the total number of LD cases reported nationwide. In this study, the annual total direct medical cost of LD cases on Maryland Eastern Shore was $1,455,081; 490 cases were in the clinically defined early or late stage of LD. Total indirect medical costs, nonmedical costs, and productivity losses were $436,949; 84 cases were clinically defined early- or late-stage LD. Therefore, in general, an LD patient (clinically defined early or late stage) costs $2,970 in direct medical costs plus $5,202 in indirect medical costs, nonmedical costs, and productivity losses. In 2002, 23,763 LD cases were reported to CDC. Hence, the estimated nationwide annual economic impact of LD and relevant complaints was ≈$203 million (in 2002 dollars). However, since LD cases reported on the basis of the surveillance case definition are believed to be underreported (*13*,*21*), this nationwide estimate is likely to be low.

We found that the average cost per LD case decreased over the study period. In LD-endemic areas, personal protection measures are frequently emphasized and insecticides are widely used (*22*). Persons in LD-endemic areas likely visit physicians more frequently whenever they have an exposure or an insect bite, and physicians attending patients from an LD-endemic area likely order serologic testing for possible LD patients and provide prompt treatment. However, our current evidence was limited in that we were only able to find a decrease in per capita cost within diagnosis groups (e.g., clinically defined early- and late-stage LD), but we could not find a shift in the number of cases from late to early stage. Therefore, we don't know what caused the decrease in average cost per LD case.

This study has certain limitations. First, we used clinical case definition (physician determination) instead of surveillance case definition of LD because of limited data. Thus, we may have overestimated the number of LD cases. As a result of case definition, our estimation of cost not only included the cost of LD (clinically defined early- and late-stage LD) but also the costs of LD-relevant complaints (suspected LD, tick bite, and other related complaints). Second, medical charges used in our study may not reflect the true cost. Third, our results are likely to underestimate the costs per case because some of the costs were not included. Costs that were omitted included any costs incurred by a patient beyond the study period. Likewise, Steer et al. reported that ≈7% of LD cases remained asymptomatic within the 20-month study (*23*). These asymptomatic patients may have costs beyond the study. Public health surveillance and administration costs and intangible costs (e.g., costs incurred because of pain and suffering) were also not incorporated in the study. Fourth, because of the large variance between mean and median costs, using mean cost to estimate national impact could be an overestimation. Finally, this study is also limited in that we only had information for indirect medical costs, nonmedical costs, and productivity losses from ≈8% of total patients in the study. Therefore, the results from survey data were extrapolated to represent the whole study population. This method may have biased our results.

LD is the most common vectorborne zoonotic inflammatory disease in the United States. The longterm sequelae of LD are debilitating to patients and costly to society. The emergence of LD and previous experience predict the feasibility of public health interventions for LD control and prevention (*24*). More research on the social behavior of LD patients and economic evaluation of LD prevention interventions is needed.

## Appendix 1

### Cost and Risk Questionnaire

Download the PDF athttp://wwwnc.cdc.gov/eid/pdfs/12/4/05-0602-Techapp.pdf.

## Appendix 2

### Direct Medical Costs Equation

For each patient, direct medical costs of Lyme disease (LD) and relevant complaints were calculated as follows:Y_i_ = Σ C_it_

Where *Y* is the total direct medical costs for patient *i* and *C_it_* is the direct medical cost of type *t* for patient *i*. Different types (*t*) of direct medical costs involved in this study included physician visits, consultation, serology, procedure, therapy, hospitalization/emergency room, and other relevant costs.

Cost of physician visits: refers to total visit charges per patient with primary provider or physician managing LD. It includes charges for procedures only performed in the office at the time of the visit. All other procedures were covered in the cost of procedure category.

Cost of consultation: refers to total charges per patient accrued at the time of consultation including only those procedures (e.g., laboratory procedures or electrocardiography) done in the office at that time. All other procedures ordered by the consult but done off site (e.g., magnet resonance imaging or computerized tomography) were covered in the cost of procedure category.

Cost of serology: refers to total charges of LD serology tests (e.g., enzyme-linked immunosorbent assay or Western blotting test) for each patient.

Cost of procedure: refers to total charges per patient of all other procedures not covered in office visits, consultation, or hospital and emergency room.

Cost of therapy: refers to total charges per patient of therapy including antibiotics and additional costs associated with intravenous therapy (e.g., other medications such as saline solutions, heparin, local anesthetics; registered nurse home visits; X-ray; etc.)

Cost of hospitalization/emergency room: refers to total hospital and/or emergency room charges per patient. It includes physician fees and ambulance fees.

Other relevant costs: refer to other appropriate charges for each patient such as charges for additional laboratory tests.

## Appendix 3

### Multivariate Regression Model

We used a multivariate linear regression model to estimate the relative impact of a number of factors on the direct medical costs of Lyme disease (LD).*Y*_i_ = *X*_i_ β + ε_i_

where *Y*_i_, the dependent variable, is the total direct medical costs for patient i and *X*_i_ is a vector of covariates. ε_i_ is a mean-zero random error. Baseline costs came from the intercept term, which refers to a female patient who had tick bite only (without erythema migrans symptoms) diagnosed in 1997 during an office visit. Such a patient had no hospital or emergency room stay, no serologic tests, no consultation from other physicians, no antimicrobial drug therapy, and no other procedures outside physician office and hospital/emergency room. Additional direct medical costs were added or subtracted to the baseline costs for each independent variable of interest if statistically significant. Therefore, total direct medical costs per LD patient = baseline costs + cost difference of different patient diagnosis group (early stage LD, late stage LD, suspected LD, other LD relevant complain vs. tick bite ) + costs of serologic test + costs of other procedure outside physician office and hospital/emergency room + costs of hospital and/or emergency room stay + costs of consultation from other physicians + costs of therapy + miscellaneous costs + cost difference with or without erythema migrans + cost difference of gender + cost difference of each additional year of age + cost difference of cohort year, if statistically significant.
